# EASIX and Severe Endothelial Complications After CD19-Directed CAR-T Cell Therapy—A Cohort Study

**DOI:** 10.3389/fimmu.2022.877477

**Published:** 2022-04-08

**Authors:** Felix Korell, Olaf Penack, Mike Mattie, Nicholas Schreck, Axel Benner, Julia Krzykalla, Zixing Wang, Michael Schmitt, Lars Bullinger, Carsten Müller-Tidow, Peter Dreger, Thomas Luft

**Affiliations:** ^1^ Department of Internal Medicine V, University Hospital Heidelberg, Heidelberg, Germany; ^2^ Department of Hematology, Oncology and Tumor Immunology, Charité-Universitätsmedizin Berlin, Corporate Member of Freie Universität Berlin, Humboldt-Universität zu Berlin, and Berlin Institute of Health, Berlin, Germany; ^3^ Kite Pharma, Gilead, Santa Monica, CA, United States; ^4^ Division of Biostatistics, German Cancer Research Center, Heidelberg, Germany

**Keywords:** CAR-T cell, EASIX, CRS, ICANS, prognostic biomarker

## Abstract

**Background:**

Endothelial dysfunction is associated with two main complications of chimeric antigen receptor T (CAR-T) cell therapy, cytokine release syndrome (CRS) and immune effector cell-associated neurotoxicity syndrome (ICANS). This study evaluates the Endothelial Activation and Stress Index (EASIX) as a prognostic marker for high-grade CRS and ICANS in patients treated with CD19-directed CAR-T cells.

**Methods:**

In this retrospective study, a training cohort of 93 patients from the ZUMA-1 trial and a validation cohort of 121 patients from two independent centers (University Hospital Heidelberg, Charité University Medicine Berlin) were investigated. The primary objective was to assess the predictive capacity of EASIX measured immediately before the start of lymphodepletion (EASIX-pre) for the occurrence of grade ≥3 CRS and/or ICANS. To explore a possible endothelial link, serum levels of endothelial stress markers (angiopoietin-2, suppressor of tumorigenicity-2, soluble thrombomodulin, and interleukin-8) were determined before lymphodepletion and on day 7 after CART infusion in the validation cohort (*n* = 47).

**Results:**

The prognostic effect of EASIX-pre on grade ≥3 CRS and/or ICANS was significant in the training cohort [OR 2-fold increase 1.72 (1.26–2.46)] and validated in the independent cohort. An EASIX-pre cutoff >4.67 derived from the training cohort associated with a 4.3-fold increased odds ratio of severe CRS/ICANS in the independent cohort. Serum endothelial distress markers measured on day+7 correlated with EASIX-pre and associated with severe complications.

**Conclusions:**

EASIX-pre is a powerful predictor of severe CRS/ICANS after CD19-directed CART therapy and might be used as a basis for risk-adapted prevention strategies.

## Introduction

T cells transduced with a CD19-directed chimeric antigen receptor (CAR) have become standard of care (SOC) for patients with B-lineage acute lymphoblastic leukemia (ALL), large B-cell lymphoma (LBCL), and mantle cell lymphoma (MCL), and have dramatically impacted treatment algorithms in these entities in recent years (1–4).

Cytokine release syndrome (CRS) is the most common side effect of CD19-directed CAR-T cell therapy. Depending on product- and patient-specific factors, CRS can occur in its severe form (grade ≥ 3) in up to 46% of the patients (5, 6). The release of cytokines after CAR-T cell activation triggers an inflammatory response that leads to constitutional symptoms such as fever and fatigue (5, 7). Severe complications are possible, such as hypotension, respiratory failure, shock, and organ dysfunction (5, 7).

Neurotoxicity—classified as immune effector cell-associated neurotoxicity syndrome (ICANS)—is another frequent complication of CD19-directed CAR-T cell therapy, occurring in up to 67% of patients, with severe and potentially lethal courses in a substantial proportion of patients (grade ≥ 3) (5, 6, 8, 9). ICANS can develop simultaneously with CRS, after CRS has ceased or independent from CRS (7). Its pathophysiology is incompletely understood; however, cytokine and chemokine release may serve as trigger as in CRS. Furthermore, CD19-expressing mural cells surrounding the endothelium in the brain could be direct targets of CAR-T (10).

Both CRS and ICANS have been linked to endothelial pathology (11–15). The increasingly recognized prominent role of endothelial dysfunction for complications of allogeneic stem-cell transplantation (alloSCT) led us to hypothesize that similar endothelial pathomechanisms may also contribute to CART-related CRS/ICANS. In particular, transplant-associated thrombotic microangiopathy (TAM) represents a life-threatening complication after alloSCT that is characterized by high lactate dehydrogenase (LDH), high creatinine, and low platelets (16, 17). In order to establish a continuous marker that predicts risk of TAM and other endothelial complications in the context of alloSCT, these three routine parameters were used for calculation of the Endothelial Activation and Stress Index (EASIX) (18, 19). EASIX is a validated predictor of endothelial complications and mortality after alloSCT and acute GvHD (18–24). Additionally, EASIX is an effective prognostic biomarker of mortality of COVID-19 (25). Recently, EASIX was reported to associate with both CRS and ICANS, and modifications of EASIX removing creatinine [simplified EASIX (s-EASIX)] or replacing creatinine by C-reactive protein (CRP, modified EASIX, m-EASIX) showed improved prediction power in a single-center study (26). Furthermore, combination of EASIX with inflammation markers (CRP and ferritin) improved prediction in another single-center study (27). So far, no validated analyses were reported.

The endothelial relationship of EASIX in the CAR-T cell setting remains to be established. Assessment of serum parameters associated with endothelial activation and distress such as ANG2 (Angiopoietin-2), ST-2 (suppressor of tumorigenicity 2), sCD141 (soluble thrombomodulin), and IL8 (interleukin 8) could help to understand endothelial contribution to the pathogenesis of CRS/ICANS.

In this study, we investigated whether EASIX is a prognostic factor for severe CRS and/or ICANS in patients receiving CD19-directed CAR-T cell therapy. Additionally, the correlation of endothelial serum markers with EASIX-pre and their association with severe CRS and/or ICANS was examined.

## Materials and Methods

### Patients and CAR-T Cell Products

Patients who had received axicabtagene ciloleucel (axi-cel) as part of the ZUMA-1 trial (ClinicalTrials.gov number: NCT02348216) (1) and gave permission to explore the results in a post-hoc analysis represented the training cohort (*n* = 93). The validation cohort consisted of 121 patients treated with CD19-directed CAR-T cells (axi-cel, tisagenlecleucel/tisa-cel, or HD-CAR-1) at the University Hospital Heidelberg (*n* = 97) and Charité University Medicine Berlin (*n* = 24) from Oct. 1, 2018, through September 30, 2021.

Axi-cel and brexucabtagene autoleucel (Brexu-cel) are autologous anti-CD19 CAR-T cell products containing a second-generation CAR encoded by a retroviral vector with an scFy targeting CD19 with CD3ζ and CD28 intracellular domains that signal T-cell activation (1, 4). Tisa-cel is generated from autologous T cells transduced with a second-generation lentiviral vector to express an anti-CD19 CAR containing a CD3ζ domain and a 4-1BB (CD137) domain as costimulatory signal (2). In the HD-CAR-1 study, autologous T cells are transduced with a third-generation retroviral CAR vector encoding for anti-CD19 with CD3ζ for T-cell activation and CD28 and 4-1BB domains as costimulatory signals. HD-CAR-1 CAR-T cells were administered in variable doses ranging from 1 × 10^6^/m^2^, 5 × 10^6^/m^2^, up to 20 × 10^6^/m^2^ body surface area after lymphodepletion (28). All patients of both the training and validation cohorts received lymphodepleting chemotherapy with fludarabine and cyclophosphamide in doses determined according to the manufacturer or study protocol (1, 2, 28).

The training cohort included 86 patients with DLBCL and 7 patients with PBMCL (1). In the Heidelberg cohort, patients were treated with standard of care Axi-cel, Tisa-cel, or HD-CAR-1 on the trial protocol. Axi-Cel was administered to 39 r/r DLBCL patients and three r/r PMBCL patients, while Tisa-Cel was administered to 15 r/r DLBCL patients and Brexu-Cel was given to three r/r MCL patients (1, 2). In the HD-CAR-1 study, CAR-T cells were administered to six r/r DLBCL patients, six r/r MCL patients, six r/r CLL patients, four r/r FL patients, and 15 ALL patients (28). All 24 patients (23 patients with DLBCL and one with ALL) from the Berlin cohort were treated with standard of care Tisa-Cel (2).

In the training cohort, patients received tumor lysis prophylaxis and prophylaxis for infection with pneumocystis pneumonia, herpes virus, and fungal infections according to NCCN guidelines or standard institutional practice (3). In the validation cohort, infection prophylaxis and supportive care were administered as described previously (29, 30). Specifically, all patients treated in Heidelberg received endothelial protection with pravastatin and ursodeoxycholic acid (UDCA) until day +14 (29).

According to the Declaration of Helsinki, written informed consent for all patients was obtained. Ethical approval and approvals from the local and federal competent authorities were granted from the Ethics Committee (Medical Faculty of Heidelberg University, reference number: AFmu-405/2017, October 2017).

### CRS/ICANS Grading and Endpoints

Severity for both CRS and ICANS was graded according to Lee et al. (ZUMA1) and ASTCT consensus criteria (validation cohort) (5, 31, 32). The following dichotomization of severity of CAR-T cell therapy complications was used for the investigation of EASIX: severe (grade ≥3) CRS and/or ICANS versus neither severe CRS nor severe ICANS.

Concomitant high-grade ICANS and CRS cannot be distinguished, in particular in patients requiring mechanical ventilation. We therefore decided to combine both grade ≥3 CRS and ICANS as primary endpoint.

### Assessment of EASIX and Serological Markers of Endothelial Activation

For the training cohort, EASIX was calculated at three different time points: EASIX before start of lymphodepletion (EASIX-pre), EASIX on the day of CAR-T administration (EASIX-d0), and EASIX on day 3 after application (EASIX-d3). In the validation cohort, EASIX-pre, EASIX-d0, EASIX-d3, and EASIX-d7 were available for analysis. Additionally, serum levels of four endothelial markers were measured in 47 patients of the Heidelberg cohort. Time of blood sampling was analogous to the EASIX measurement before lymphodepletion, as well as on day 7. Samples were stored at −80°C and ST-2, ANG2, sCD141, and CXCL8 were analyzed using commercial ELlSA (DuoSet**^®^** ELISA, Biotechne R&D, USA) according to the manufacturer**’**s descriptions.

### Statistical Analysis

EASIX was calculated by the formula: LDH (U/L) × creatinine (mg/dl)**/**platelets (10^9^ cells/L). A binary logarithm (log2)-transformed index, log2(EASIX-pre), was used for the primary statistical analysis. The EASIX derivatives were calculated by the formula: LDH (U/L)**/**platelets (10^9^ cells/L) (sEASIX), and by the formula: LDH (U/L) × CRP (mg/dl)**/**platelets (10^9^ cells/L) (mEASIX).

For the combination of both types of events (CRS and/or ICANS), an optimized univariate cutoff of EASIX-pre was estimated both by Generalized Maximally Selected (Chi-Squared) Statistics and a Conditional Inference Tree with EASIX-pre as the only splitting variable (33, 34). This cutoff was graphically confirmed by a partial dependence plot using multivariate conditional inference forests that besides EASIX-pre also includes established confounders [age, gender, disease (aggressive B-cell lymphoma vs. others) and disease stage (refractory disease and progressive disease vs. others)].

Distributions of baseline data were described by standard statistical measures, number, frequency, and Fisher tests for categorical variables and median, range (minimum/maximum), interquartiles with 25th and 75th percentiles (IQ), and Kruskal–Wallis tests for continuous data. Uni- and multivariable binary logistic regression analyses provided odds ratios (OR) for the prognostic effect of EASIX-pre (continuous log2 transformed or with cutoff) on risk of grade ≥3 complications. Covariables were age, gender, disease (aggressive B-cell lymphoma vs. others), and disease stage (refractory disease and progressive disease vs. others). As only Axi-cel was given in the training cohort, the product could not be included as confounder. These multivariate prognostic effects were validated in the validation cohort by comparing the prediction accuracy of the full model, a nested reduced model (without EASIX) and a reference model (intercept only), all fitted based on the training cohort, *via* Brier scores estimated on the validation cohort. Explorative multivariate models in the validation cohort used the Firth correction because of rare events in the gender distribution. ROC curve analyses (including area under the curve, AUC) were used to assess the prediction performance of EASIX scores as univariate predictors. Additionally, Pearson correlations were used for associations of (log)EASIX with (log) endothelial serum markers. Univariate logistic regression was used to assess the effect of endothelial serum markers on the risk of grade ≥3 associations.

All calculations were performed using R version 4.0.4 (R Foundation for Statistical Computing, Vienna, Austria, 2021). In all tests, a *p*-value < 0.05 was considered significant without corrections for multiple testing.

## Results

### Patient Characteristics

Training and validation cohorts were comparable in terms of age and gender. There was also no significant difference of disease status before start of the procedure, with the vast majority of patients entering lymphodepletion with unresponsive disease. Due to the inclusion of HD-CAR-1 study patients and those treated with SOC tisa-cel in the validation cohort, however, distribution of underlying diagnoses was significantly different. Detailed patient characteristics are given in **Table 1**.

**Table 1:** Baseline characteristics, CRS, ICANS, and EASIX.

	Training cohort (ZUMA1-trial; *n* = 93)	CAR-T validation cohort (Heidelberg and Berlin; *n* = 121)	*p*-values
**Date of CAR-T cell application**	November 1, 2015, to September 30, 2016	Oct 1, 2018, to September 30, 2021	
**Median age at CAR-T application (years)**	58 (23–76)	60 (20–83)	0.78
**Patient sex**			0.55
Female	30 (32%)	34 (28%)	
Male	63 (68%)	87 (72%)	
**Disease**			<0.001
ALL	–	16 (13%)	
NHL	93 (100%)	105 (87%)	
DLBCL	86 (92%)	83 (69%)	
MCL	–	9 (7%)	
CLL	–	6 (5%)	
FL	–	4 (3%)	
PMBCL	7 (8%)	3 (2%)	
**CAR-T cell product**			<0.001
Axicabtagene ciloleucel	93 (100%)	42 (35%)	
Tisagenlecleucel	0	39 (32%)	
HD-CAR-1	0	37 (31%)	
Brexucabtagene autoleucel	0	3 (2%)	
**Disease status before lymphodepletion**			0.003
CR	8 (9%)	7 (6%)	
PR/MRD	1 (1%)	16 (13%)	
SD	12 (13%)	17 (14%)	
PD	71 (76%)	80 (66%)	
NA	1 (1%)	1 (1%)	
**CRS or ICANS (CRS/ICANS)**			<0.001
No CRS or ICANS	6 (7%)	51 (42%)	
CRS or ICANS 1–2	55 (59%)	49 (41%)	
CRS 1–2	74 (80%)	60 (50%)	
ICANS 1–2	34 (37%)	15 (12%)	
CRS or ICANS ≥ 3	32 (34%)	21 (17%)	
Onset of CRS or ICANS (days)	2 (1–12)	4 (0–14)	<0.001
**EASIX (Median, IQ25/75)**			
EASIX-pre	1.8 (0.3–106.1, IQ 1.0/4.7)	2.2 (0.3–97.7, IQ 1.2/4.0)	0.38
EASIX-d0	2.0 (0.3–120.4, IQ 1.1/4.1)	2.0 (0.3–91.5, IQ 1.2/4.2)	0.96
EASIX-d3	2.8 (0.3–57.9, IQ 1.7/6.2)	2.4 (0.3–69.1, IQ 1.4/4.9)	0.13
EASIX-d7	NA	2.5 (0.4–94.0, IQ 1.5/6.7)	–

EASIX, Endothelial Activation and Stress Inde; CAR, chimeric antigen receptor; ALL, acute lymphoblastic leukemia; NHL, non-Hodgkin lymphoma; DLBCL, diffuse large B-cell lymphoma; MCL, mantle cell lymphoma; CLL, chronic lymphocytic leukemia; FL, follicular lymphoma; PMBCL, primary mediastinal B-cell lymphoma; HD-CAR-1, Heidelberg Chimeric Antigen Receptor Trial 1; CR, complete remission; PR, partial remission; SD, stable disease; PD, progressive disease; CRS, cytokine release syndrome; ICANS, immune effector cell-associated neurotoxicity syndrome; IQ = interquartile [25/75, Q1/Q3 (lower and upper quartile)]; NA, not available.

EASIX-pre training n = 90, validation n = 121; EASIX-d0 training n = 85, validation n = 121; EASIX-d3 training n = 85, validation n = 121; EASIX-d7 training not applicable, validation n = 121.

### Frequency of CRS and ICANS

In the training cohort, 83 (90%) experienced CRS while ICANS was observed in 62 (67%) patients. Grade ≥ 3 CRS was detected in 9 patients (10%) with a median onset of 2 (1–12) days and high-grade ICANS occurred in 28 patients [30%; median onset 6 (1–17) days]. A total of 87 patients (93%) developed either CRS or ICANS or both (grade ≥3: 32 patients, 34%).

Of the 121 patients of the validation cohort, 70 patients (58%) developed CRS grades 1–4 and 29 patients (24%) developed ICANS grades 1–4. Higher-grade CRS (grade ≥ 3) affected 10 patients (8%) with a median onset of 4 (0–14) days, while grade ≥ 3 ICANS occurred in 14 patients [12%; median onset 6 (3–17) days]. A total of 70 patients (58%) experienced either CRS or ICANS or both (grade ≥ 3: 21 patients, 17%) (**Table 1**).

### EASIX Before and After CART-Infusion

There were no significant differences between training and validation cohorts regarding EASIX scores measured at any time point. Similar EASIX trends were measured over time in subgroups of patients with and without aggressive B-cell lymphoma (**Supplementary Figure 1A**). Age groups below and above 60 years differed in EASIX-pre in the training cohort, and in EASIX-d3 and d7 in the validation cohort only, with higher values in older patients (**Supplementary Figure 1B**). Patients with progressive or refractory disease had higher EASIX values than patients with stable or sensitive disease at any time point in the validation cohort, but not in the training cohort (**Supplementary Figure 1C**).

### Association of EASIX with Severe CRS/ICANS

Prognostic value of EASIX before chemotherapy was tested in the training cohort by multivariate logistic regression including age, gender, diagnosis, and disease status as confounding variables (**Table 2**). EASIX-pre associated with grade ≥3 CRS/ICANS after CART infusion with an odds ratio of 1.71 (*p*-value < 0.001). This model was validated in the independent cohort using the Brier score. The model including EASIX-pre had an improved prediction performance (Brier score 0.151) in comparison to the reference model (0.173) and the multivariable model without EASIX (0.166).

**Table 2:** Multivariable logistic regression, classifier CRS/ICANS ≥3, training cohort (*n* = 90, events = 32).

Binary Endpoint: CRS/ICANS ≥ 3	OR (95% CI)	*p*-value
**EASIX-pre (per log2)**	1.72 (1.26–2.46)	0.001
Age (per 10 years)	1.06 (0.69–1.66)	0.783
Gender (male vs. female)	0.90 (0.31–2.73)	0.850
Diagnosis (aggr. B-cell lymphoma vs. other)	1.48 (0.42–5.92)	0.558
Disease status at lymphodepletion	1.52 (0.49–5.24)	0.478

EASIX ,Endothelial Activation and Stress Index; pre, prior lymphodepletion; log, logarithm; CRS, cytokine release syndrome; ICANS, immune effector cell-associated neurotoxicity syndrome; OR, odds ratio; 95% CI, 95 percent confidence interval.

Diagnosis patients with aggressive B-cell lymphoma vs. other. Disease status: patients with progressive or refractory disease vs. patients with stable disease or response (complete or partial).

**Figure 1:**
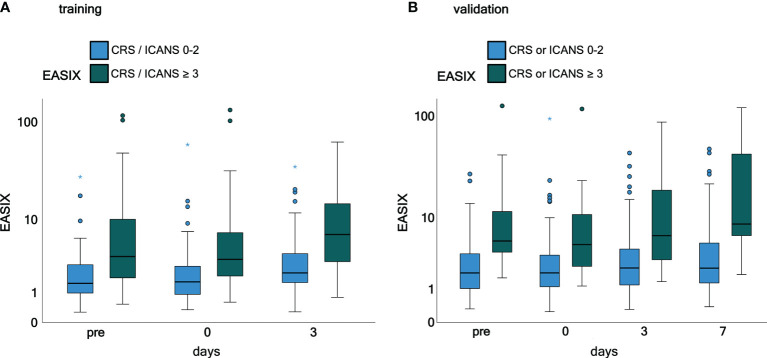
EASIX measurement in the training and validation cohorts grouped according to CRS and ICANS grade. **(A)** Training cohort. **(B)** Validation cohort. Patients were grouped according to CRS and/or ICANS grade. EASIX prior lymphodepletion on the day of CAR-T cell application and on day 3 and 7 after application is shown. Training cohort: CRS and/or ICANS grades 0-2 (*n* = 61) and ≥ 3 (*n* = 32), validation cohort: CRS and/or ICANS grades 0-2 (*n* = 100) and ≥ 3 (*n* = 21). EASIX, Endothelial Activation and Stress Index; pre, prior lymphodepletion; CRS, cytokine release syndrome; ICANS, immune effector cell-associated neurotoxicity syndrome.

**Figure 2:**
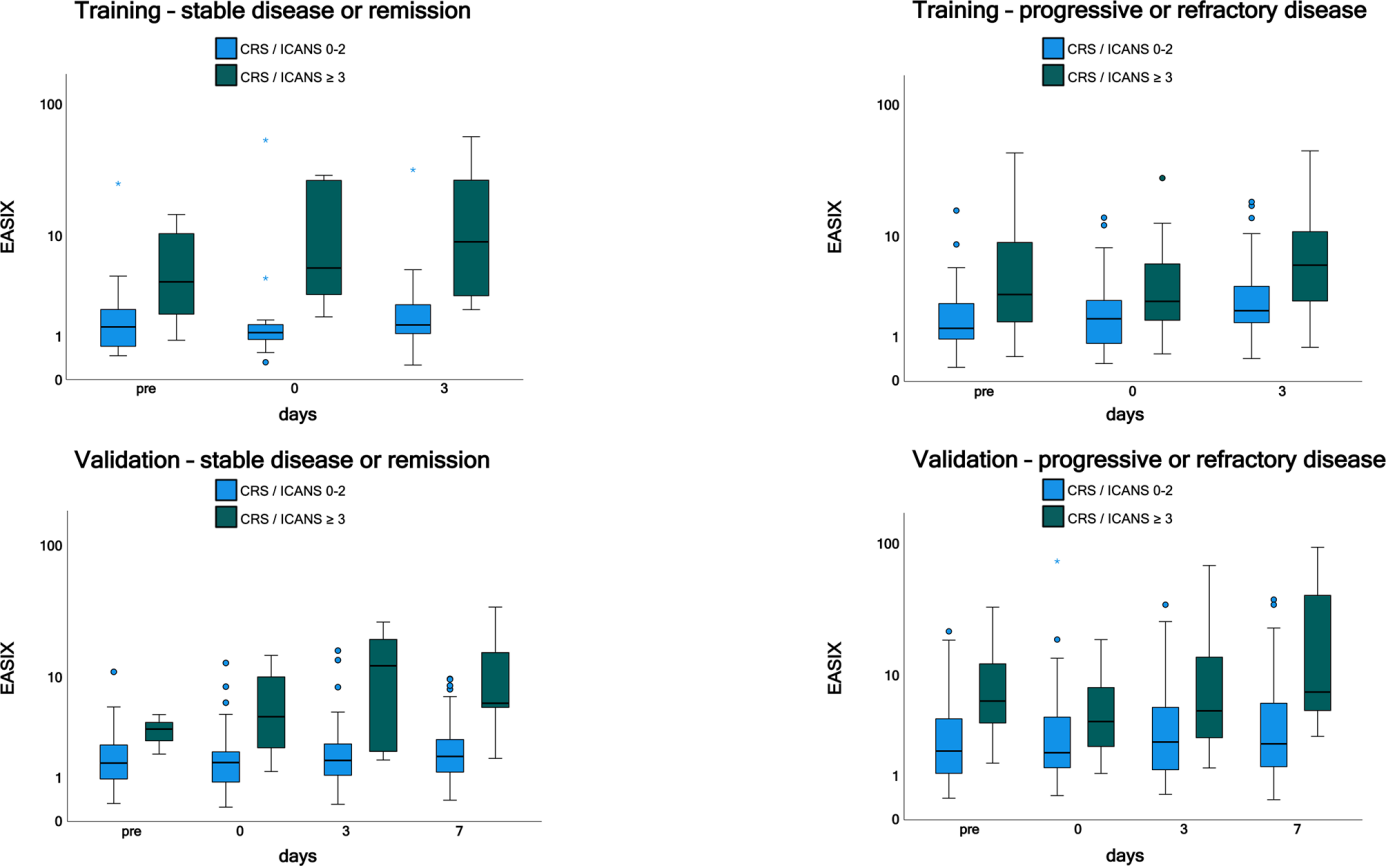
EASIX measurement in the training and validation cohorts by disease status. Patients with progressive or refractory disease and patients with stable or sensitive disease were grouped according to CRS and/or ICANS grades 0-2 and ≥ 3. EASIX prior lymphodepletion, on the day of CAR-T cell application and on day 3 and 7 after application is shown. EASIX, Endothelial Activation and Stress Index; pre, prior lymphodepletion; CRS, cytokine release syndrome; ICANS, immune effector cell-associated neurotoxicity syndrome.

Time courses of EASIX in patients who have experienced or will experience grade ≥3 CRS or ICANS events throughout the observation period are visualized in **Figure 1**. EASIX values were increased in patients of both cohorts with (future) complications at any time points. A similar picture was observed in patients with progressive or refractory disease status at lymphodepletion (**Figure 2**), as well as in patients in remission/stable disease (not shown).

The three single EASIX parameters were analyzed individually in separate explorative models (**Supplementary Figures 2A, B** and **Supplementary Tables 1, 2**). Whereas there was a correlation between LDH-pre and grade ≥3 CRS/ICANS in both cohorts, and also measurable, but inconsistent effects of creatinine and platelets determined before lymphodepletion, CRP levels at lymphodepletion had no impact.

### EASIX Derivatives: sEASIX and mEASIX

**Figure 3:**
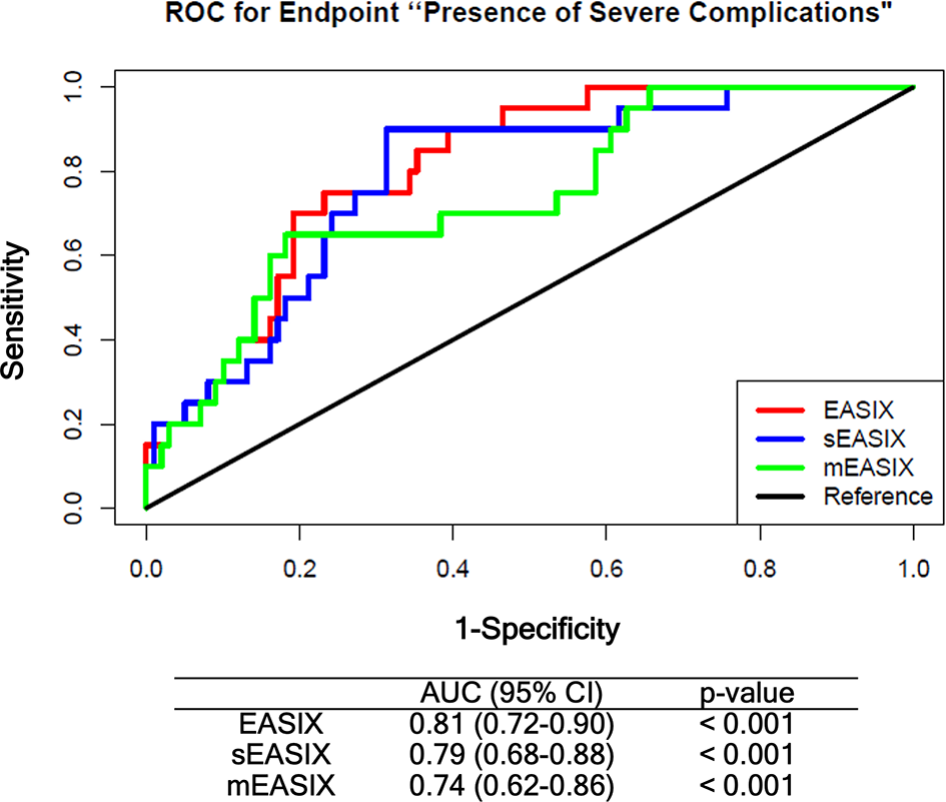
ROC analysis comparing EASIX-pre, sEASIX, and mEASIX as prognostic marker of severe complications. Validation of the prediction performance of univariate models. All models were fitted based on the training cohort (ZUMA, *n* = 79) and validated in the Heidelberg/Berlin cohort (*n* = 119) (respective subpopulations with available CRP data). EASIX, Endothelial Activation and Stress Index; sEASIX, simplified EASIX; mEASIX, modified EASIX; ROC, receiver operating characteristic; AUC, area under the curve; 95% CI, 95 percent confidence interval.

Prognostic values of EASIX derivatives before chemotherapy were tested in the training cohort by multivariate logistic regression including age, gender, diagnosis, and disease status as confounding variables (**Supplementary Tables 3, 4**). Both derivatives associated with grade ≥3 CRS/ICANS after CART infusion with an odds ratio of 1.63, *p*-value = 0.004 (sEASIX) and 1.22, *p*-value = 0.015 (mEASIX). Validation of univariate simplified (s)EASIX and modified (m)EASIX models calculated in the training cohort showed that both EASIX derivatives did not compare favorably to EASIX-pre in ROC analysis (**Figure 3**). Validation of multivariable models revealed that mEASIX showed lower prediction errors [Brier score model with confounders (no EASIX) 0.184, model with EASIX 0.173, with sEASIX 0.173, mEASIX 0.155]. Confining the analysis to Axi-cel-treated patients, we calculated Brier scores in the validation cohort (*n* = 45) for the model with confounders 0.211, model with EASIX 0.176, with sEASIX 0.179, mEASIX 0.180.

### Validation of Optimized EASIX Cutoff

The maximally selected Chi-Squared statistic revealed three potential univariate cutoff values for EASIX-pre in the training cohort (3.09, 4.67, and 5.73). The conditional inference tree with EASIX as the only splitting variable splits the dataset exactly once at the cutoff 4.67. Graphical validation based on the partial dependence plot of EASIX-pre in a multivariate conditional inference forest (including all other confounders as potential splitting variables as well) supported the cutoff 4.67 as the optimal value in the training cohort.

Comparing the prediction performance of a multivariate model including the derived EASIX cut point, a model with only the confounders, and a reference model with the intercept only, prediction errors were smallest in the model with EASIX cutoff 4.67 (Brier score 0.162 vs. 0.166 with confounders only, vs. 0.173 reference model).

When testing the derived cutoff in a newly fitted multivariate model, EASIX-4.67 constitutes a prognostic factor for severe complications with an OR of 4.3 (1.5–12.7, *p* = 0.006) (**Table 3**), which can be interpreted as further statistical validation of the prognostic effect of EASIX-pre cut 4.67 for severe CRS/ICANS.

**Table 3:** Multivariable logistic regression (Firth correction) with the EASIX cutoff (categorial) (Validation cohort, *n* = 121, 21 events).

Binary Endpoint: CRS/ICANS ≥ 3	OR (95% CI)	*p*-value
**EASIX-pre (cutoff >4.67)**	4.32 (1.52–12.67)	0.006
Age (per 10 years)	0.93 (0.66–1.35)	0.709
Gender male vs. female	4.56 (1.27–24.81)	0.018
Diagnosis (aggr. B-cell lymphoma vs. other)	1.07 (0.35–3.68)	0.905
Disease status at lymphodepletion	1.50 (0.50–4.91)	0.478

EASIX, Endothelial Activation and Stress Index; pre, prior lymphodepletion; log, logarithm; CRS, cytokine release syndrome; ICANS, immune effector cell-associated neurotoxicity syndrome; OR, odds ratio; 95% CI, 95 percent confidence interval.

Diagnosis patients with aggressive B-cell lymphoma vs. other. Disease status: patients with progressive or refractory disease vs. patients with stable disease or response (complete or partial).

Looking at the two evolving subgroups in an unadjusted manner, 11/94 (11.7%) patients had severe complications in the subgroup with EASIX<4.67, compared to 10/27 (37%) patients in the high EASIX subgroup (*p* = 0.0071). For patients treated with Axi-cel, 7/35 (20%) developed severe CRS/ICANS in the low EASIX subgroup, whereas 6/10 (60%) developed such events in the high EASIX subgroup (*p* = 0.022).

Finally, patients in the EASIX-pre low-risk group (<4.67) who did not develop severe CRS/ICANS until day+7 had a very low risk of developing these complications subsequently (3/83, 3.5% in the validation cohort).

### Endothelial Serum Markers and Grade ≥3 CRS/ICANS

**Figure 4:**
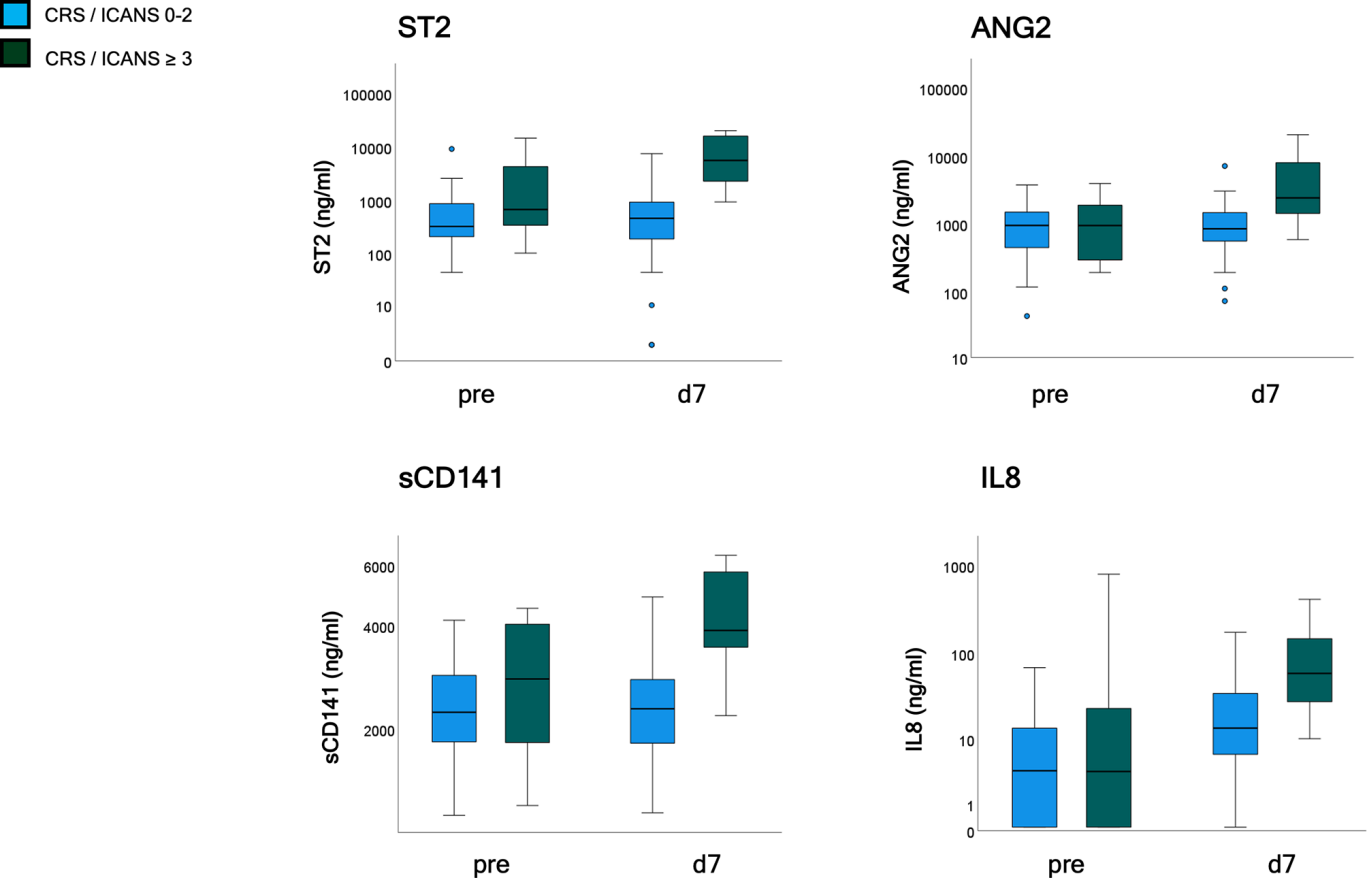
Endothelial serum marker kinetics (validation cohort, *n* = 47). Patients were grouped according to CRS and ICANS grade with analysis of endothelial markers (ST2, ANG2, sCD141, and IL8) prior to lymphodepletion and on day 7 after application. CRS and/or ICANS grades 0-2 (*n* = 39) and ≥ 3 (*n* = 8). EASIX, Endothelial Activation and Stress Index; ST2, suppressor of tumorigenicity 2; ANG2, Angiopoietin-2; sCD141, soluble thrombomodulin; IL8, interleukin 8; pre, prior lymphodepletion; d, day.

Serum markers of endothelial distress were measured in 47 patients of the validation cohort before and after CAR-T cell infusion. ST2 and sCD141 showed a weak, ANG2, and CXCL8 no correlation with EASIX-pre when assessed before lymphodepletion, but all four markers moderately correlated with EASIX-pre when assessed on day+7 (**Supplementary Table 5**). Univariable logistic regression analyses revealed an association with severe complications for ST2-pre [OR per log2 1.49 (0.98–2.26), *p* = 0.061], and no association for the other markers (ANG2-pre *p* = 0.986, sCD141-pre *p* = 0.341; IL-8-pre *p* = 0.676). In contrast, marker values measured on day+7 were associated with severe CRS/ICANS occurring before or after day 7 for all four serum markers (ORs for doubling of marker values): ST2-d7 OR 2.9 (1.7–5.2), *p* < 0.001; ANG2-d7 OR 2.9 (1.5–5.2), *p* = 0.001; sCD141-d7 OR 13.4 (3.0–59.8), *p* = 0.001; IL-8-d7 OR 1.9 (1.2–2.9), *p* = 0.004 (**Figure 4**).

## Discussion

CRS and ICANS are frequent complications of CAR-T cell therapy, which—in their severe form—contribute to morbidity and mortality associated with this type of cellular immunotherapy. Because of their side effects and their potential negative impact on CAR-T cell efficacy, aggressive prevention strategies, such as prophylactic or preemptive administration of anti-inflammatory agents should be reserved for those patients in need for them. Thus, identification of reliable indicators of an increased risk of developing these complications is of paramount importance.

The EASIX score has proven its suitability as a predictor of various endothelial complications and mortality after alloSCT, but also in other complex settings of endothelial activation or damage, such as COVID-19 (18–25). Given the accumulating evidence that endothelial pathomechanisms are involved in the pathogenesis of CRS and ICANS (11–15), the EASIX score is an obvious candidate for being explored as biomarker in this context. To this end, two recent papers by Pennisi et al. and Greenbaum et al. reported that EASIX measured before lymphodepletion could predict the risk of higher grade CRS (26, 27). In addition, in the Greenbaum study, EASIX-pre predicted also grade 2-4 ICANS (27), whereas increases of the EASIX score following axi-cel treatment heralded both severe CRS and ICANS in the Pennisi study (26). The present analysis confirmed and for the first time validated the capacity of EASIX-pre to predict the risk of CRS/ICANS in two additional cohorts treated with axi-cel and a variety of CD19-directed CAR-T cell products.

In contrast to the Pennisi study, modifications of the EASIX formula by omitting creatinine (sEASIX) or replacing it with CRP (mEASIX) did not result in increased prediction power on univariable ROC analysis compared to the original EASIX-pre in the present study, although prediction errors were lower for mEASIX in multivariable logistic regression. Anyway, the differences between the 3 formulas raised before lymphodepletion were marginal in the two datasets tested here as well as in the Pennisi sample.

We could demonstrate here that high EASIX before CART infusion was paralleled by significant increases of a variety of endothelial serum markers after CART, thereby extending preliminary evidence reported by Gust et al. for grade 4–5 ICANS (11). This supports the notion of endothelial involvement in the pathogenesis of CRS/ICANS and proves EASIX as a practical prognostic marker of endothelial dysfunction also in the CART setting.

Similar to the situation before alloSCT, correlation between EASIX-pre and the endothelial biomarkers measured before lymphodepletion and biomarker association with severe CRS/ICANS, respectively, were only weak or absent. This does not rule out a pre-existing endothelial pathology as a driver of the predictive capacity of EASIX-pre since it may reflect hidden endothelial defects not captured by the standard biomarker repertoire. This interpretation would be in line with preliminary data from the Gust study suggesting increased pre-lymphodepletion ratios of angiopoietin 2 to angiopoietin 1 in patients with grade 4–5 ICANS (11).

Practical implications of this work consist primarily in the perspective of risk-adapted prevention strategies. These could include more aggressive consideration of novel prophylactic or pre-emptive anti-inflammatory strategies, such as early use of steroids or IL-1 receptor antagonists (35, 36). Moreover the EASIX-pre score could be considered in decision-making on CAR-T-cell therapy indication in patients with critical comorbidities (37). On the other hand, EASIX-pre could be an important tool for designing CAR-T-cell outpatient strategies.

A limitation of this study is the retrospective/post-hoc nature of the analyses. The training cohort only included axi-cel-treated patients who suffered higher rates of severe complications, and product-specific validation of EASIX effects was therefore not possible in this study. Furthermore, although grades 3–4 are similarly defined by severe hypoxia and vasopressor-dependent hypotension, CRS in the ZUMA-1 study was not graded according to current consensus criteria. The analyses are correlative and causal relations cannot be claimed.

Strengths of the study consist in a straightforward training/validation design using independent patient cohorts and in the comprehensive correlative biomarker work-up.

In conclusion, this study adds evidence for endothelial involvement in CRS/ICANS following CAR-T cell treatment of B-lymphoid malignancies. More importantly, this work validates EASIX-pre as a robust prognostic marker of severe endothelial complications across a variety of CD19-based CAR-T cell products and indications. Because it is easy to use and instantly available, it appears as an ideal tool for developing risk-adapted prevention strategies.

## Data Availability Statement

The original contributions presented in the study are included in the article/**Supplementary Material**. Further inquiries can be directed to the corresponding author.

## Ethics Statement

The studies involving human participants were reviewed and approved by the Medical Faculty of Heidelberg University, reference number: AFmu-405/2017, October 2017. The patients/participants provided their written informed consent to participate in this study.

## Author Contributions

FK and TL designed the research project and analyzed the data. FK, TL, OP, LB, MM, and ZW acquired the data. NS, AB, JK, MS, and TL analyzed the data. NS, PD, and FK discussed the data and the organization of the manuscript. FK, NS, AB, JK, and TL wrote the manuscript. All authors critically reviewed the manuscript. MS, OP, LB, CM-T, and PD edited the manuscript. TL supervised the work. All authors contributed to the article and approved the submitted version.

## Funding

TL was supported by Deutsche Krebshilfe 70113520. OP acknowledges the support of José Carreras Leukämie-Stiftung (3R/2019, 23R/2021), Deutsche Krebshilfe (70113519), Deutsche Forschungsgemeinschaft (PE 1450/7-1, PE 1450/9-1) and StiftungCharité BIH (BIH_PRO_549, Focus Group Vascular Biomedicine).

## Conflict of Interest

Authors MM and ZW were employed by the company Kite Pharma, Gilead.

The remaining authors declare that the research was conducted in the absence of any commercial or financial relationships that could be construed as a potential conflict of interest.​

## Publisher’s Note

All claims expressed in this article are solely those of the authors and do not necessarily represent those of their affiliated organizations, or those of the publisher, the editors and the reviewers. Any product that may be evaluated in this article, or claim that may be made by its manufacturer, is not guaranteed or endorsed by the publisher.

## Supplementary Material

Supplementary Figure 1 EASIX measurement in the training and validation cohorts at different timepoints – subgroup analysis. **(A)** Disease type: subgroup analysis of patients with and without aggressive B cell lymphoma. **(B)** Age: subgroup analysis of age groups with patients below 60 years and patients 60 years or older. **(C)** Disease status: subgroup analysis of patients with progressive or refractory disease and patients with stable disease or response (complete or partial).

Supplementary Figure 2 Single EASIX parameters (LDH, creatinine, platelets) measurement in the training and validation cohorts at different timepoints in subgroups of patients with and without grade ≥3 CRS, ICANS or both CRS and ICANS. **(A)** training cohort **(B)** validation cohort. Patients were grouped according to CRS and ICANS grade with analysis of LDH, creatinine and platelets prior lymphodepletion, on the day of CAR-T cell application and on day 3 and 7 after application. LDH, lactate dehydrogenases; pre, prior lymphodepletion; CRS, cytokine release syndrome; ICANS, immune effector cell-associated neurotoxicity syndrome.

Supplementary Figure 3 CRP measurement in the training and validation cohorts at different timepoints in subgroups of patients with and without grade ≥3 CRS, ICANS or both CRS and ICANS. **(A)** training cohort **(B)** validation cohort. Patients were grouped according to CRS and ICANS grade with analysis of CRP prior lymphodepletion, on the day of CAR-T cell application and on day 3 and 7 after application. CRP, C-reactive protein; pre, prior lymphodepletion; d, day; CRS, cytokine release syndrome; ICANS, immune effector cell-associated neurotoxicity syndrome.

Supplementary Figure 4 Asymptotic Generalized Maximally Selected Statistics. We applied maximally selected statistics in order to investigate whether we can define a cut-point for the variable “EASIXpre” in univariate logistic regression with end point severe grade ≥3 CRS and/or ICANS. Best cut-point is EASIX≤ 4.67, p=0.005.
