# Supplementary material for: EASIX and Severe Endothelial Complications After CD19-Directed CAR-T Cell Therapy—A Cohort Study
Source: Front Immunol. 2022 Apr 8;13:877477. doi: 10.3389/fimmu.2022.877477 (PMC9033201; doi:10.3389/fimmu.2022.877477)
Supplement: Supplementary Figure 1 — EASIX measurement in the training and validation cohorts at different timepoints – subgroup analysis. (A) Disease type: subgroup analysis of patients with and without aggressive B cell lymphoma. (B) Age: subgroup analysis of age groups with patients below 60 years and patients 60 years or older. (C) Disease status: subgroup analysis of patients with progressive or refractory disease and patients with stable disease or response (complete or partial). [file DataSheet_1.pdf]

Supplemental Figure 1A.

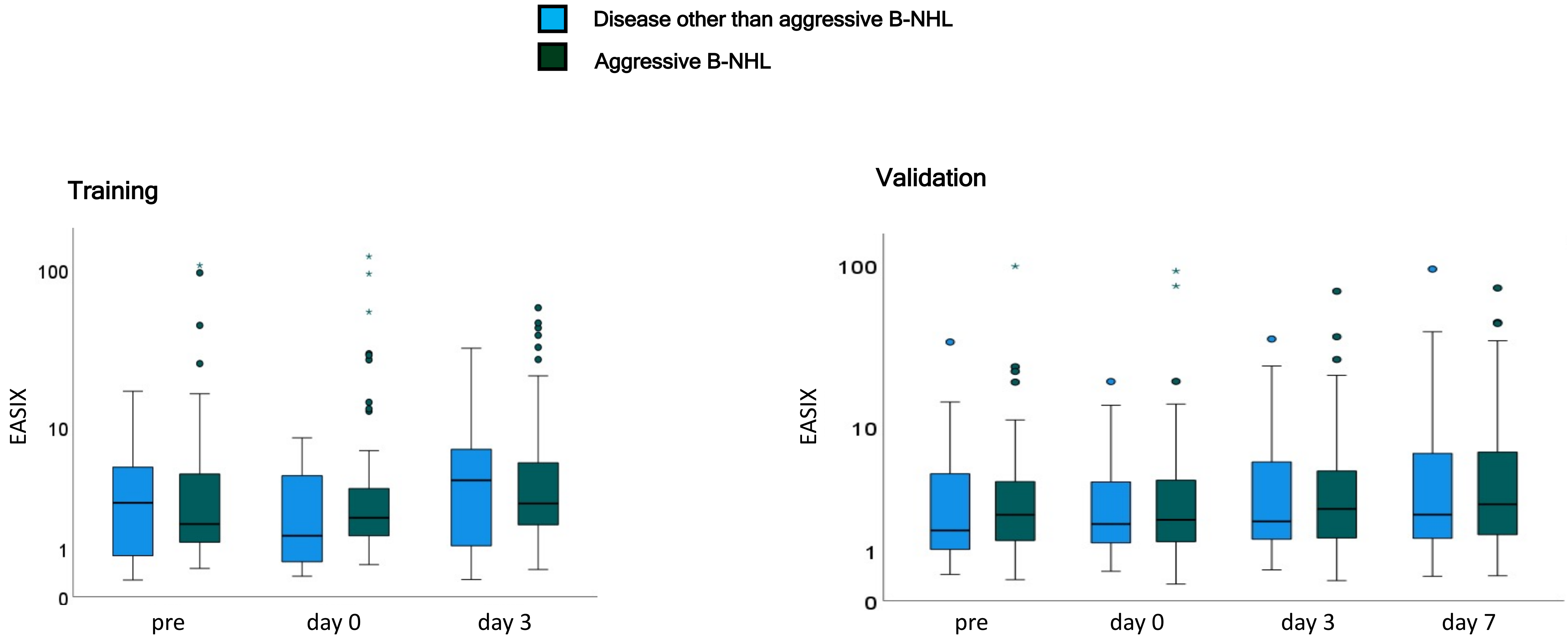

Supplemental Figure 1B.

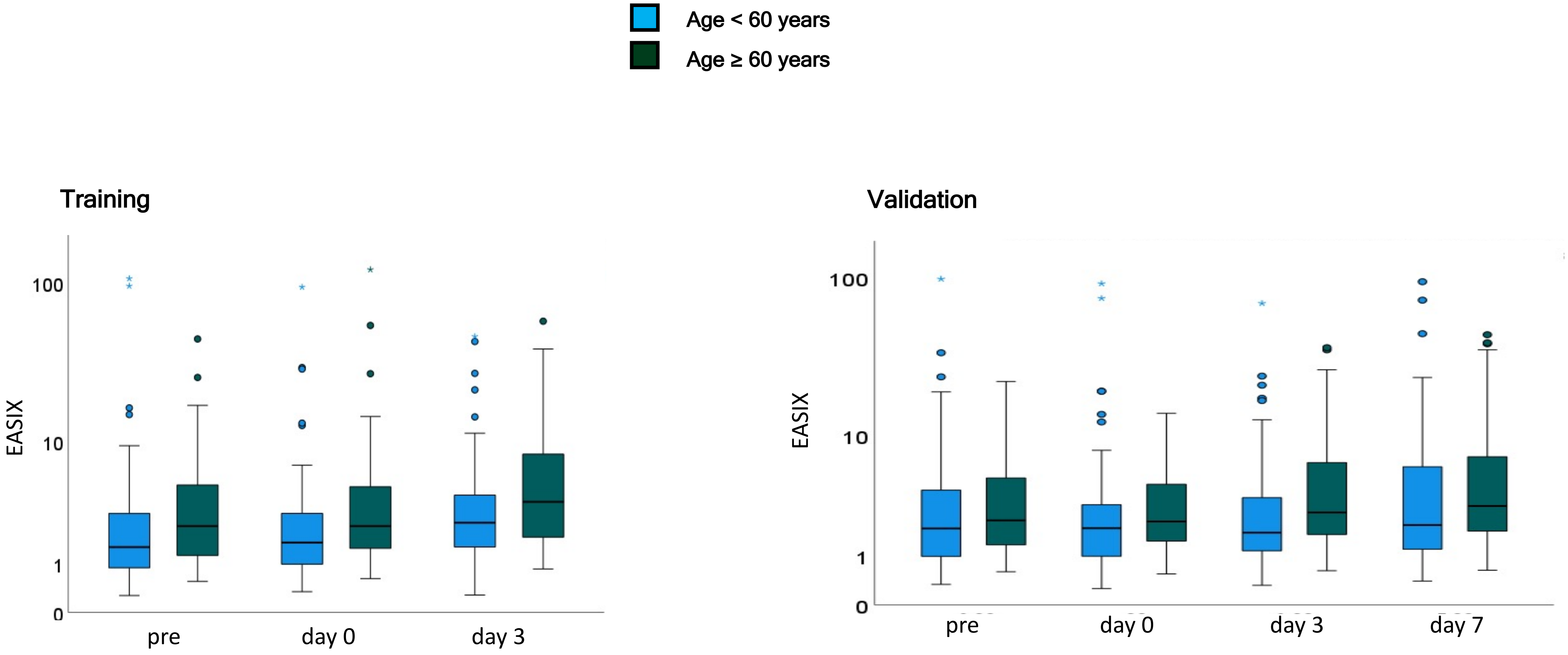

Supplemental Figure 1C.

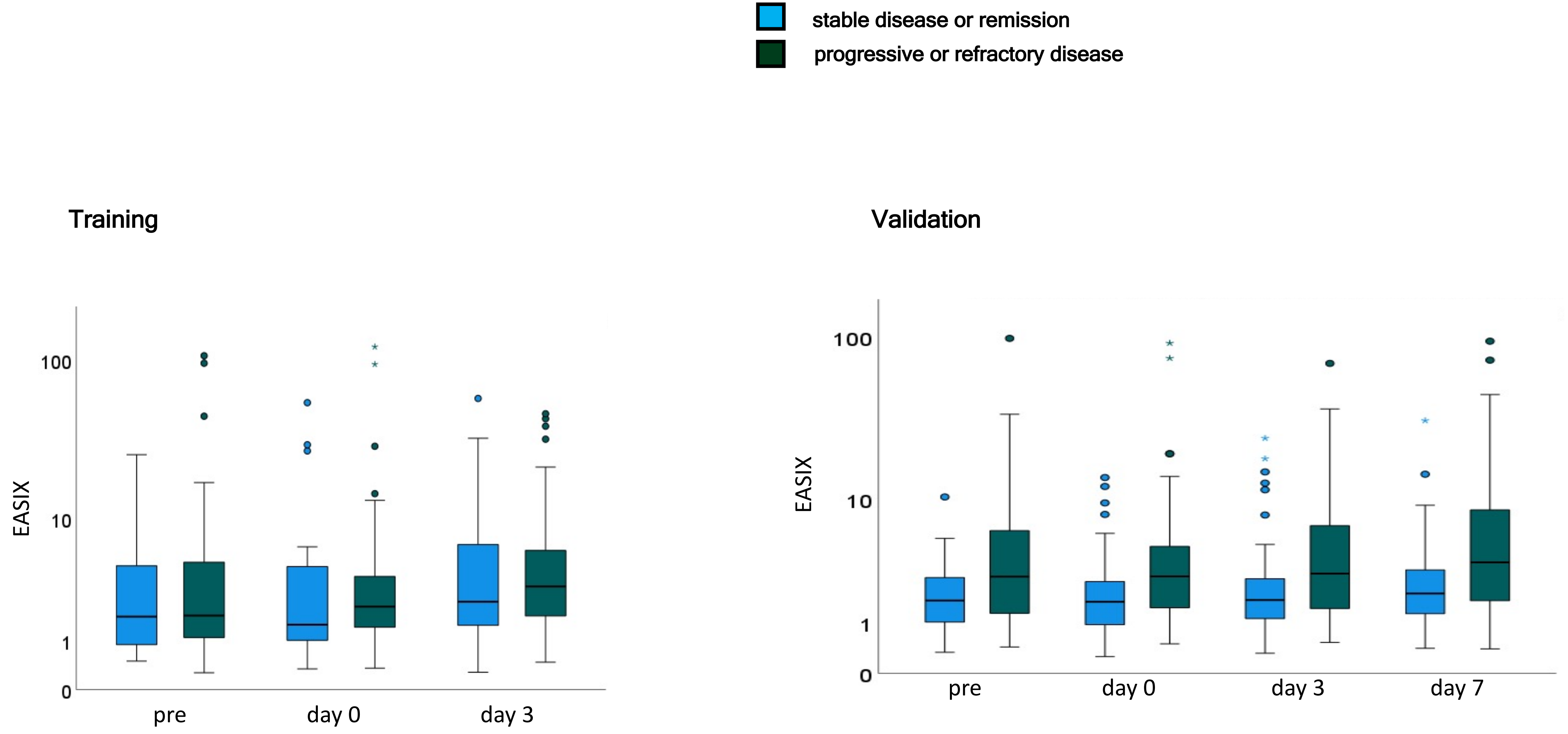

Supplement figure 2A (training).

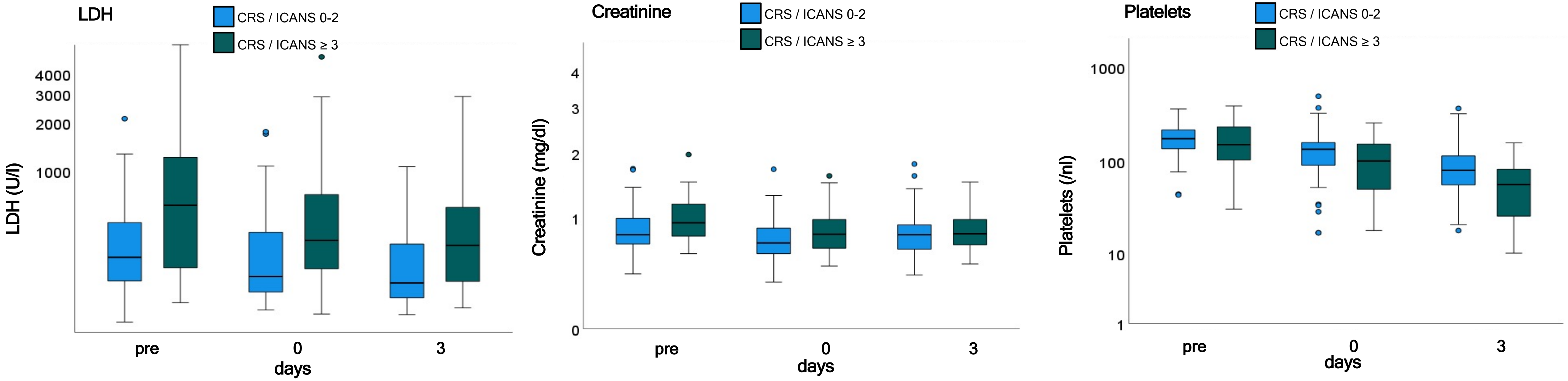

Supplement figure 2B (validation).

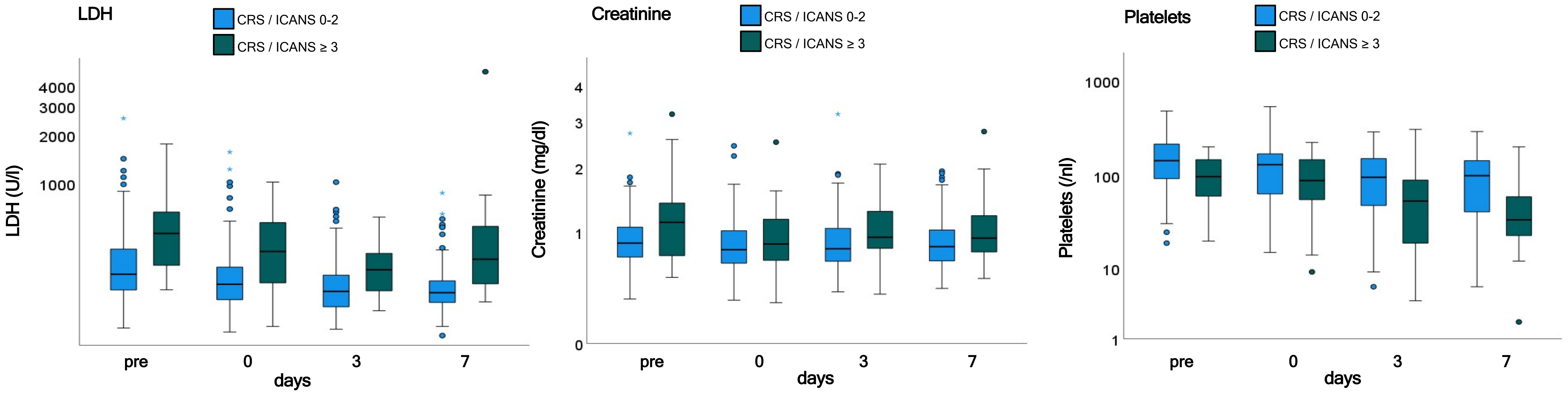

Supplement Figure 3A (training).

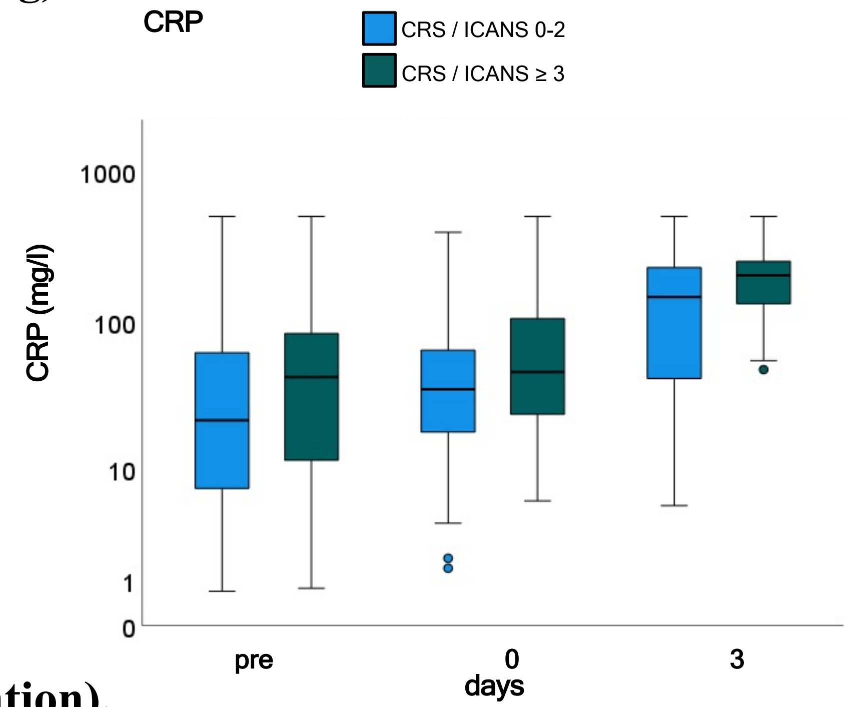

Supplement Figure 3B (validation).

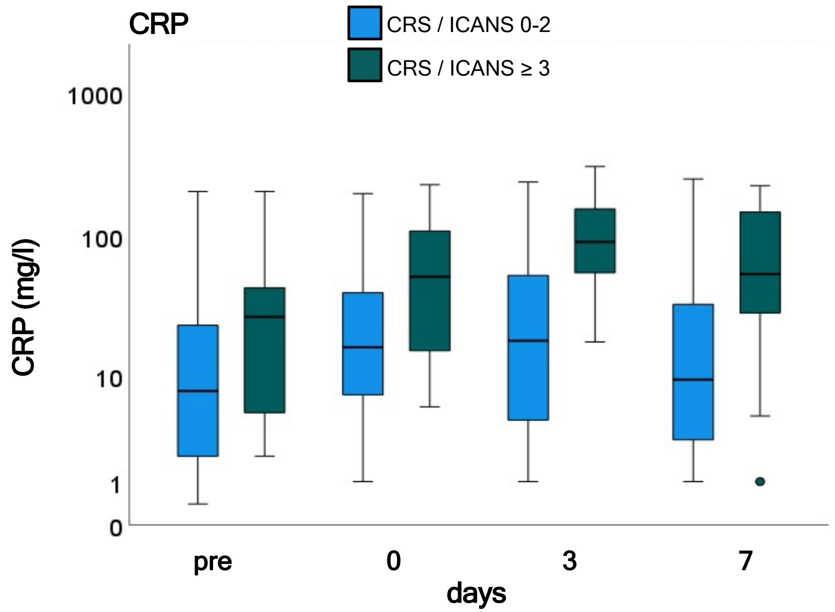

Supplement Figure 4

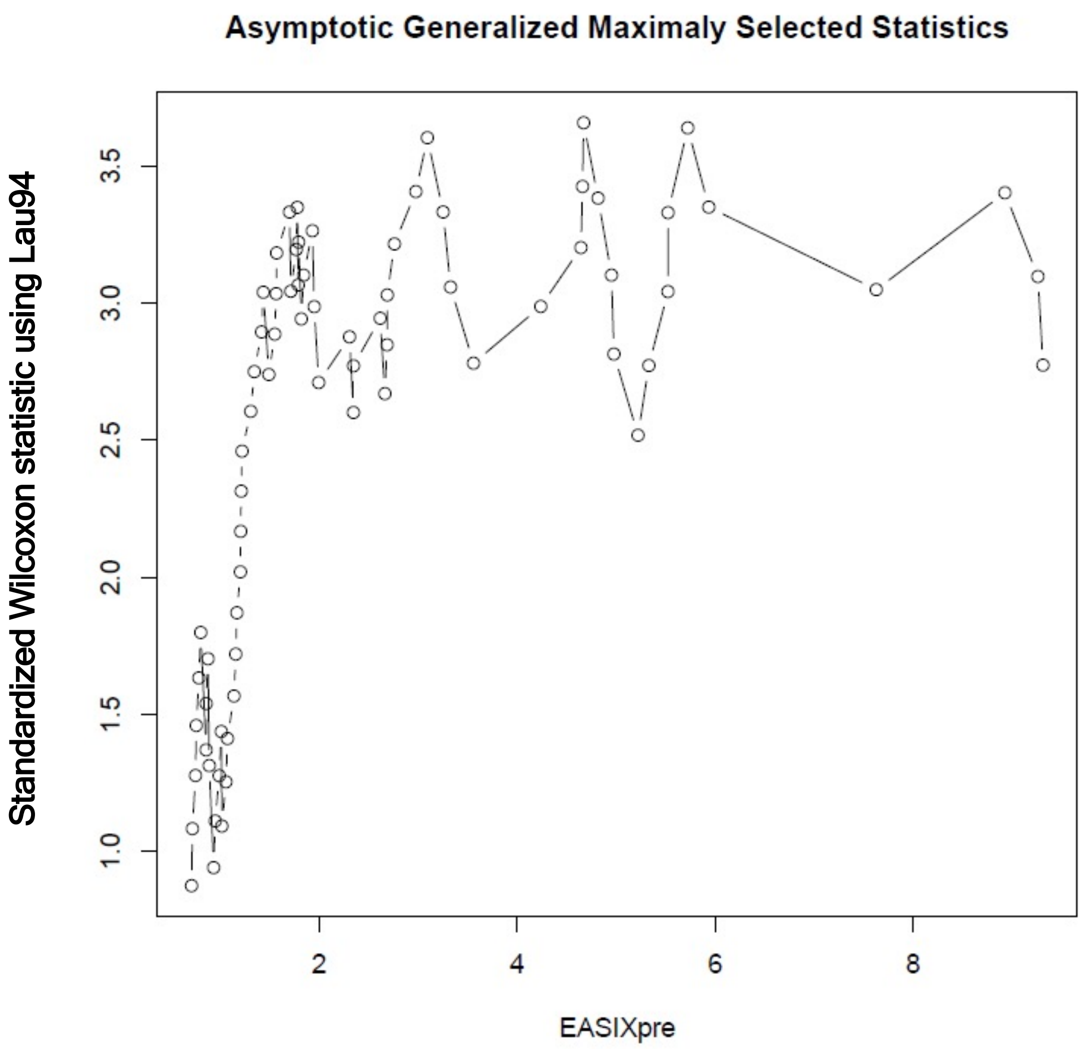

Suppl. Table 1: Single EASIX parameters and CRP in the training cohort (n= 79, events=30)

Binary Endpoint: CRS/ICANS ≥ 3	OR (95% CI)	p-value
**LDH-pre (per log2)**	1.98 (1.17-3.59)	0.016
**Creatinine-pre (per log2)**	5.06 (1.28-25.79)	0.032
**Platelets-pre (per log2)**	0.89 (0.35-2.25)	0.812
**CRP-pre (per log2)**	1.01 (0.79-1.28)	0.957
Age (per 10 years)	1.03 (0.62-1.72)	0.914
Gender (male vs female)	0.58 (0.15-2.10)	0.402
Diagnosis (aggr. B-cell lymphoma vs other)	1.21 (0.31-5.29)	0.794
Disease status at lymphodepletion	0.85 (0.21-3.65)	0.820

LDH = Lactate dehydrogenase. CRP = C-reactive protein. log = logarithm. pre = prior lymphodepletion. CRS = cytokine release syndrome. ICANS = immune effector cell-associated neurotoxicity syndrome. OR = odds ratio. 95% CI = 95 percent confidence interval.
Diagnosis patients with aggressive B cell lymphoma vs. other. Disease status: patients with progressive or refractory disease vs patients with stable disease or response (complete or partial).

Suppl. Table 2: Single EASIX parameters and CRP in the validation cohort (n= 119, events=21)

Binary Endpoint: CRS/ICANS ≥ 3	OR (95% CI)	p-value
**LDH-pre (per log2)**	2.53 (1.20-5.87)	0.019
**Creatinine-pre (per log2)**	1.93 (0.57-7.11)	0.302
**Platelets-pre (per log2)**	0.42 (0.20-0.79)	0.011
**CRP-pre (per log2)**	0.99 (0.69-1.39)	0.938
Age (per 10 years)	1.03 (0.68-1.62)	0.886
Gender (male vs female)	5.13 (1.04-41.68)	0.072
Diagnosis (aggr. B-cell lymphoma vs other)	1.34 (0.34-6.62)	0.692
Disease status at lymphodepletion	1.00 (0.27-3.86)	0.998

LDH = Lactate dehydrogenase. CRP = C-reactive protein. log = logarithm. pre = prior lymphodepletion. CRS = cytokine release syndrome. ICANS = immune effector cell-associated neurotoxicity syndrome. OR = odds ratio. 95% CI = 95 percent confidence interval.
Diagnosis patients with aggressive B cell lymphoma vs. other. Disease status: patients with progressive or refractory disease vs patients with stable disease or response (complete or partial).

Suppl. Table 3 - Multivariate logistic analysis for the training (ZUMA) population (s-EASIX score)
(n= 90, events=32)

Binary Endpoint: CRS/ICANS ≥ 3	OR (95% CI)	p-value
**sEASIX-pre (per log2)**	1.63 (1.19-2.33)	0.004
Age (per 10 years)	1.09 (0.72-1.68)	0.686
Gender (male vs female)	1.01 (0.35-2.99)	0.989
Diagnosis (aggr. B-cell lymphoma vs other)	1.52 (0.45-5.94)	0.518
Disease status at lymphodepletion	1.44 (0.48-4.80)	0.529

s-EASIX = simplified Endothelial Activation and Stress Index. pre = prior lymphodepletion. log = logarithm. CRS = cytokine release syndrome. ICANS = immune effector cell-associated neurotoxicity syndrome. OR = odds ratio. 95% CI = 95 percent confidence interval.
Diagnosis patients with aggressive B cell lymphoma vs. other. Disease status: patients with progressive or refractory disease vs patients with stable disease or response (complete or partial).

Suppl. Table 4 - Multivariate logistic analysis for the training (ZUMA) population (m-EASIX score)
(n= 79, events=30)

Binary Endpoint: CRS/ICANS ≥ 3	OR (95% CI)	p-value
**mEASIX-pre (per log2)**	1.22 (1.05-1.44)	0.015
Age (per 10 years)	1.13 (0.74-1.74)	0.577
Gender (male vs female)	0.71 (0.23-2.17)	0.540
Diagnosis (aggr. B-cell lymphoma vs other)	1.04 (0.32-3.58)	0.954
Disease status at lymphodepletion	1.45 (0.41-5.69)	0.576

m-EASIX = modified Endothelial Activation and Stress Index. pre = prior lymphodepletion. log = logarithm. CRS = cytokine release syndrome. ICANS = immune effector cell-associated neurotoxicity syndrome. OR = odds ratio. 95% CI = 95 percent confidence interval.
Diagnosis patients with aggressive B cell lymphoma vs. other. Disease status: patients with progressive or refractory disease vs patients with stable disease or response (complete or partial).

Suppl. Table 5 - Association of EASIX and EASIX parameters prior lymphodepleting chemotherapy with endothelial distress markers on different timepoints before and after CAR-T cell application

	Pearson-correlation (95% CI)
**EASIX-pre**	
ST2-pre	0.26 (-0.03; 0.51)
ST2-d0	0.19 (-0.08; 0.44)
ST2-d3	0.44 (0.12; 0.68)
ST2-d7	0.48 (0.28; 0.65)
ANG2-pre	-0.05 (-0.33; 0.24)
ANG2-d0	0.29 (0.02; 0.52)
ANG2-d3	0.51 (0.20; 0.72)
ANG2-d7	0.56 (0.37; 0.71)
sCD141-pre	0.26 (-0.03; 0.51)
sCD141-d0	0.57 (0.35; 0.73)
sCD141-d3	0.55 (0.25; 0.75)
sCD141-d7	0.55 (0.36; 0.70)
IL8-pre	0.10 (-0.19; 0.38)
IL8-d0	0.17 (-0.10; 0.42)
IL8-d3	0.19 (-0.16; 0.50)
IL8-d7	0.51 (0.30; 0.67)
**LDH-pre**	
ST2-pre	0.20 (-0.10; 0.46)
ST2-d0	0.12 (-0.15; 0.38)
ST2-d3	0.19 (-0.17; 0.50)
ST2-d7	0.31 (0.08; 0.51)
ANG2-pre	-0.10 (-0.38; 0.20)
ANG2-d0	0.08 (-0.19; 0.35)
ANG2-d3	0.19 (-0.16; 0.50)
ANG2-d7	0.28 (0.04; 0.48)
sCD141-pre	0.13 (-0.17; 0.40)
sCD141-d0	0.34 (0.07; 0.56)
sCD141-d3	0.42 (0.09; 0.67)
sCD141-d7	0.24 (-0.00; 0.45)
IL8-pre	0.13 (-0.17; 0.40)
IL8-d0	0.05 (-0.22; 0.32)
IL8-d3	0.03 (-0.31; 0.37)
IL8-d7	0.23 (-0.01; 0.44)
**Creatinine-pre**	
ST2-pre	-0.16 (-0.43; 0.14)
ST2-d0	-0.01 (-0.28; 0.26)
ST2-d3	0.26 (-0.09; 0.55)
ST2-d7	0.17 (-0.08; 0.39)
ANG2-pre	-0.22 (-0.48; 0.07)

ANG2-d0	0.08 (-0.19; 0.35)
ANG2-d3	0.23 (-0.13; 0.53)
ANG2-d7	0.20 (-0.04; 0.42)
sCD141-pre	0.37 (0.10; 0.60)
sCD141-d0	0.53 (0.30; 0.70)
sCD141-d3	0.38 (0.04; 0.64)
sCD141-d7	0.32 (0.08; 0.52)
IL8-pre	-0.16 (0.42; 0.14)
IL8-d0	-0.01 (-0.28; 0.27)
IL8-d3	-0.08 (-0.41; 0.28)
IL8-d7	0.20 (-0.04; 0.42)
**Platelets-pre**	
ST2-pre	-0.29 (-0.54; -0.01)
ST2-d0	-0.22 (-0.47; 0.05)
ST2-d3	-0.40 (-0.65; -0.07)
ST2-d7	-0.41 (-0.59; -0.19)
ANG2-pre	-0.15 (-0.42; 0.15)
ANG2-d0	-0.35 (-0.57; -0.09)
ANG2-d3	-0.52 (-0.73; -0.21)
ANG2-d7	-0.55 (-0.70; -0.35)
sCD141-pre	-0.03 (-0.31; 0.26)
sCD141-d0	-0.33 (-0.55; -0.07)
sCD141-d3	-0.32 (-0.59; 0.03)
sCD141-d7	-0.49 (-0.65; -0.28)
IL8-pre	-0.12 (-0.40; 0.17)
IL8-d0	-0.24 (-0.48; 0.03)
IL8-d3	-0.38 (-0.61; 0.01)
IL8-d7	-0.50 (-0.66; -0.29)

EASIX = Endothelial Activation and Stress Index. ST2 = suppressor of tumorigenicity 2. ANG2 = Angiopoietin-2. sCD141 = soluble thrombomodulin. IL8 = interleukin 8. pre = prior lymphodepletion. d = day.
